# Editorial: Current trends in oral surgery and implantology

**DOI:** 10.3389/froh.2026.2005603

**Published:** 2026-10-16

**Authors:** Alessandro Antonelli, Karol Ali Apaza Alccayhuaman, Francisco Correia

**Affiliations:** 1 Department of Health Sciences, Magna Graecia University of Catanzaro, Catanzaro, Italy; 2 Biomaterials and Technology, Department of Research, University Center for Dental Medicine Basel UZB, University of Basel, Basel, Switzerland; 3 Specialization in Periodontology and Implants, Faculty of Dental Medicine, University of Porto, Porto, Portugal

**Keywords:** biomaterials, dental implants, digital workflow, minimally invasive surgery, oral surgery, patient-reported outcomes, regenerative dentistry

Editorial on the Research Topic Current trends in oral surgery and implantology

## Introduction

Oral surgery and implantology remain among the most dynamic fields in modern dentistry, influenced by continuous developments in biomaterials, digital planning, and surgical techniques (1, 2). Over recent decades, implant therapy has evolved from a predominantly empirical practice into a highly predictable technology-driven science. In parallel, regenerative oral surgery has extended the boundaries of what is clinically achievable in patients with complex anatomical or oncological disease (1, 3). Despite this progress, several challenges persist: ensuring and maintaining long-term implant stability, minimizing surgical complications, refining digital workflows, and customizing treatment to increasingly complex clinical scenarios, from severe bone atrophy to post-oncological reconstruction (3, 4).

This Research Topic, “Current Trends in Oral Surgery and Implantology,” was launched to document this evolving landscape, bringing together original research, systematic reviews, and case reports that evidence the improvements of contemporary clinical and translational work in this field. The contributions are organized into three main, interconnected areas: digital planning and implant stability, minimally invasive and regenerative surgical strategies, and complex implant-based rehabilitation together with the patient-centered outcomes. This collection provides a timely, multidisciplinary overview of the current state of the specialty.

## Digital precision and implant stability

Advancements in digital technologies and a deeper understanding of implant biomechanics are crucial for enhancing surgical precision. Salviati et al. describe a fully digital workflow for dental autotransplantation, reporting high surgical accuracy in a prospective cohort, while Schwärzler et al. evidence, in a preclinical micro-CT study, that milled surgical guides outperform freehand placement of orthodontic implants. The significance of implant-level factors is equally emphasized: Alagl and Madi show the antibacterial potential of biomimetic hydroxyapatite–functionalized titanium surfaces against *Staphylococcus aureus* and *Escherichia coli*, and Li et al. characterize how implant material and lingual inclination affect stress distribution in narrow-diameter posterior implants—consistent with broader evidence that macro-design and preparation technique jointly determine primary stability (5).

Stability itself is analyzed by Alvitez-Temoche et al., who investigate heat generation during osteotomy across drill diameters and implant systems, and by Al-Hity et al., who monitor the resonance-frequency stability of a one-piece conometric implant system over a six-month healing period. In terms of evidence synthesis, Arbildo-Vega et al. show, in an umbrella review, that short implants perform similarly to standard-length implants, offering a less invasive option in atrophic ridges—a scenario for which case-series evidence also supports subperiosteal implants as a viable alternative when even short implants are not feasible (6). Perioperative protocols also represent a relevant aspect. Survey data on antibiotic prophylaxis prescribing patterns among clinicians (7) highlight the importance of procedural standardization, suggesting that successful implant outcomes rely not only on device design but also on appropriate perioperative management. Together, these contributions highlight implant success from surface chemistry and macro-design, through surgical execution, to long-term comparative performance.

## Minimally invasive and regenerative strategies

A second cluster of contributions highlights the growing shift toward biologically driven, tissue-sparing strategies aimed at minimizing surgical morbidity while preserving therapeutic effectiveness. Omer et al. synthesize current evidence on the role of third molars in mandibular fractures, providing a broader perspective on their clinical and surgical implications. In parallel, growing attention to less invasive surgical approaches is reflected in recent studies showing reduced postoperative swelling with piezosurgery-assisted third molar removal (8) and supporting piezoelectric surgery as a bone-preserving option for the conservative management of odontogenic tumors (9). A similar biologically oriented perspective characterizes regenerative procedures. Taymour et al. explore the role of oral stem cells combined with hydrogels for periodontal tissue engineering, Almutiri et al. confirm the efficacy of platelet-rich fibrin for oroantral communication closure, and Madi et al. compare two deproteinized bone graft materials for socket preservation, both supporting new bone formation. This regenerative framework is further supported by a narrative review of the lateral-window technique for maxillary sinus lift grafting (10), by a randomized trial demonstrating successful osseointegration of implants placed in composite, sinus-grafted bone (11), and by a histomorphometric clinical study documenting antrostomy healing following sinus floor elevation (12). Across these studies, minimally invasive procedures and biologically active adjuncts emerge as complementary strategies for reducing surgical trauma while maintaining predictable clinical outcomes.

## Complex rehabilitation and patient-centered evidence

A further group of contributions focuses on implant-based rehabilitation in particularly challenging clinical settings. Attia et al. report on the relationship between fibula free flap complications and implant outcomes, Ci et al. describe management of thick skin flaps after fibula flap reconstruction, and Chatzidou et al. present an implant-assisted partial denture guided by the neutral zone concept after fibular reconstruction. These studies highlight how implant dentistry has become an integral part of multidisciplinary rehabilitation after head and neck oncological surgery, extending beyond implant placement itself to encompass soft-tissue management and prosthetic rehabilitation.

From a functional and patient perspective, Ye et al. compare chewing efficiency between zygomatic and conventional implant-supported restorations, an area in which careful three-dimensional radiographic assessment of malar bone engagement remains important for predictable surgical planning (13). In addition of this functional perspective, Ozgur et al. validate the Implant Treatment Experience and Knowledge Scale (ITEKS), a new patient-reported outcome measure for implant therapy. At a broader level, Shenoy et al. review four decades of literature on the causes of implant failure and available retrieval techniques, placing current clinical practice within the accumulated evidence of the field. Together, these contributions show that clinical success cannot be measured by survival rates alone, but must also account for how patients experience treatment and how the field consolidates its accumulated evidence.

## Emerging technologies: dynamic navigation and robotic-assisted surgery

Beyond the advances in digital workflows and implant stability, dynamic navigation and robotic-assisted systems are increasingly transforming the planning and execution of implant surgery. Dynamic navigation provides real-time, continuously updated visual guidance during osteotomy preparation and implant placement, while robotic-assisted platforms incorporate haptic guidance or automated control of the surgical handpiece, further minimizing operator-dependent variability. A recent systematic review comparing navigation and robot-assisted implant placement systems evidenced that robot-guided techniques achieved the smallest linear deviations, while dynamic navigation showed comparable linear accuracy and lower angular deviations in several studies. However, the limited comparative clinical evidence does not yet support definitive conclusions regarding the superiority of either approach (14). In line with this, a recent overview of dynamic and robotic computer-assisted implant surgery found that both approaches achieve clinically acceptable implant placement accuracy, with robotic systems often providing slightly greater precision. However, high costs, longer setup times, and a demanding learning curve remain important barriers to their widespread clinical adoption (15). As these technologies continue to evolve, they are likely to complement rather than replace static guided workflows by providing greater real-time adaptability, particularly in complex or anatomically challenging cases.

## Future perspectives

Taken together, the contributions to this Research Topic reflect a field advancing along several converging lines: digital precision, biologically informed materials, regenerative adjuncts, and a more complete, patient-centered definition of surgical success. As oral surgery and implant dentistry become more closely connected with digital technologies and regenerative medicine, the next challenge will be to translate these advances into robust and reproducible clinical evidence. Beyond demonstrating technical feasibility, future research should determine which approaches are most appropriate for specific clinical conditions and patient needs. Prospective and comparative studies are needed to validate emerging digital workflows and biomaterials, while greater attention should be given to standardized patient-reported outcomes and to the prevention and management of complications in complex clinical scenarios.
